# Upregulation of leucine-rich alpha-2 glycoprotein: A key regulator of inflammation and joint fibrosis in patients with severe knee osteoarthritis

**DOI:** 10.3389/fimmu.2022.1028994

**Published:** 2022-12-07

**Authors:** Ashish Sarkar, Debolina Chakraborty, Vijay Kumar, Rajesh Malhotra, Sagarika Biswas

**Affiliations:** ^1^ Council of Scientific and Industrial Research (CSIR)-Institute of Genomics and Integrative Biology, Delhi University, Delhi, India; ^2^ Academy of Scientific and Innovative Research (AcSIR), Ghaziabad, Uttar Pradesh, India; ^3^ All India Institute of Medical Sciences, New Delhi, India

**Keywords:** osteoarthritis, leucine-rich alpha-2 glycoprotein, fibrosis, biomarker, inflammation, SWATH, iTRAQ

## Abstract

**Introduction:**

Osteoarthritis (OA) is a degenerative disease of the joints mainly affecting older individuals. Since the etiology behind the progression of OA is not well understood, several associated consequences, such as synovial joint stiffness and its progression due to joint fibrosis, are still poorly understood. Although a lot of developments have been achieved in the diagnosis and management of OA, synovial fibrosis remains one of the major challenging consequences. The present study was therefore focused on understanding the mechanism of synovial fibrosis, which may further contribute to improving symptomatic treatments, leading to overall improvements in the treatment outcomes of patients with OA.

**Methods:**

We used advanced proteomic techniques including isobaric tag for relative and absolute quantitation and sequential window acquisition of all theoretical mass spectra for the identification of differentially expressed proteins in the plasma samples of patients with OA. An *in silico* study was carried out to evaluate the association of the identified proteins with their biological processes related to fibrosis and remodeling of the extracellular matrix (ECM). The most significantly upregulated protein was then validated by Western blot and enzyme-linked immunosorbent assay. The target protein was then further investigated for its role in inflammation and joint fibrosis using an *in vitro* study model.

**Results:**

Leucine-rich alpha-2 glycoprotein (LRG1) was found to be the most highly differentially expressed upregulated (9.4-fold) protein in the plasma samples of patients with OA compared to healthy controls. The knockdown of LRG1 followed by *in vitro* studies revealed that this protein promotes the secretion of the ECM in synovial cells and actively plays a role in wound healing and cell migration. The knockdown of LRG1 further confirmed the reduction of the inflammatory- and fibrosis-related markers in primary cells.

**Conclusion:**

LRG1 was identified as a highly significant upregulated protein in the plasma samples of patients with OA. It was found to be associated with increased fibrosis and cell migration, leading to enhanced inflammation and joint stiffness in OA pathogenesis.

## Introduction

Osteoarthritis (OA) is the most common inflammatory joint disorder that causes joint disability in older people, leading to poor lifestyle and increased socioeconomic burden (1, 2). Current understanding indicates that OA is a complex disease rather than just a disease affecting the cartilage only in older age. Changes due to cartilage degradation and joint fibrosis severely affect the function of the joints, leading to restrictions in movements (3). Generally, radiographic changes such as narrowing of the joint space and the formation of osteophytes are widely used to diagnose the disease due to the lack of prognostic and diagnostic markers (4). More recently, additional modalities such as magnetic resonance imaging (MRI), ultrasound (US), and optical coherence tomography (OCT) have been found to be helpful in improving the diagnosis of OA (4, 5).

Tissue fibrosis, which occurs in the synovial tissue of patients with OA, has been recently reported in other diseases such as liver, kidney, and heart diseases, leading to respective organ failure (6). Joint fibrosis is characterized by an excessive accumulation of connective tissues, resulting into moderate to severe joint stiffness, restricting the synovial joint movement and flexibility (7). It has been reported that synovial fibrosis occurs when the damaged articular cartilage is unable to repair itself and deposition of the extracellular matrix (ECM) takes place (8). The ECM is broadly regulated by transforming growth factor beta (TGF-β), which affects chondrocyte dedifferentiation, leading to the formation of fibrocartilage and resulting in joint fibrosis (3, 8, 9). The early diagnosis of OA is therefore important for clinical decision-making and the timely initiation of available therapies in order to examine the progression of tissue fibrosis. To the best of our knowledge, studies on blood-based prognostic and diagnostic markers, along with crosstalk with fibrosis, may help in understanding the fibrosis-related biological process and in the identification of novel approaches to intercept the disease progression (4, 10). Thus, biological samples such as venous blood were preferred in our study for the identification of protein-based biomarkers due to less variability and the minimally invasive procedure involved (11, 12). The OA patients included in this study opted for either total knee replacement (TKR) or unicompartmental knee replacement (UKR). In TKR, the whole knee joint was replaced, whereas only the medial compartment of the knee was replaced by prosthesis in UKR, with both cruciate ligaments and the two knee compartments being preserved. Therefore, patients who underwent UKR benefitted from a normal gait and better knee flexion compared to those who had TKR. When the disease is limited to the anteromedial compartment, UKR is a suitable option. However, TKR has been considered more durable, requires less review, and is the treatment of choice when all the ligaments and compartments are damaged (13). All of the patients who had TKR and UKR included in this study had Kellgren and Lawrence grade 4 (KL-4) OA.

Proteomics and its applications have emerged as promising techniques for the identification and characterization of clinically significant novel biomarkers from biological samples in order to understand the pathogenesis of diseases (14). In this study, we used sequential window acquisition of all theoretical mass spectra (SWATH-MS) and isobaric tag for relative and absolute quantitation (iTRAQ) to identify the differentially expressed proteins (DEPs) from the plasma samples of patients with OA and compared these to samples from healthy controls (HC) (15, 16). The most significant DEPs were then validated using Western blot (WB) and enzyme-linked immunosorbent assay (ELISA).

Out of several differential DEPs, LRG1 was identified as one of the most significantly upregulated proteins in the plasma samples of patients with OA. Reports have shown a significant link between LRG1 and fibrosis of the liver and kidney; however, its role in OA has not been evaluated yet (17–19). LRG1 is a circulatory plasma protein synthesized by hepatocytes. In collagen-induced arthritis (CIA) mice, the upregulation of LRG1 attenuated the arthritic index and fibrosis through enhancing the TGF-β/Smad2 signaling pathway (19, 20). In another study, LRG1 was observed to play a vital role in angiogenesis and mesenchymal stem cell (MSC) migration in anterior cruciate ligament transection (ACLT) mice (21). LRG1 was also shown to be linked with ocular angiogenesis and the wound healing process, which shares a similar fundamental molecular mechanism to fibrosis *via* the upregulation of LRG1 (17, 22). Previously, LRG1 was found to be upregulated and associated with inflammatory and autoimmune diseases such as systemic juvenile idiopathic arthritis and lupus nephritis (23, 24). The upregulation of LRG1 was also shown to regulate ischemia injury by promoting apoptosis and autophagy *via* the TGF-β/Smad1–5 signaling pathway (25). In the present study, for the first time, we identified and evaluated the role of LRG1 in the pathogenesis of OA and validated its role in joint fibrosis.

## Methods

### Patients and volunteers

All patients recruited in this study fulfilled the American College of Rheumatology criteria (26) for the diagnosis of knee OA. Clinically diagnosed patients with OA who were on nonsteroidal anti-inflammatory drugs (NSAIDs) (e.g., aspirin, ibuprofen, and diclofenac) and recommended to undergo either TKR or UKR as a treatment option were included in the study. Pregnant women, alcoholic patients, and patients with other diseases such as diabetes, cardiovascular disease, or any other inflammatory diseases were excluded. Biopsy synovium samples were collected from patients who had undergone surgery (TKR or UKR) as a treatment option. All of the OA patients included in this study had KL-4 score. Age-matched healthy volunteers with no history of joint pain and no clinical history of other chronic diseases were included as healthy controls (HCs). A signed written consent was also obtained from all participants of this study.

### Sample collection

Venous whole blood samples (~3.00 ml) were collected from the participants (OA patients = 60, HCs = 40) in an ethylenediaminetetraacetic acid (EDTA)-coated vacutainer (P-tech, New Delhi, India). Synovial fluid (SF; ~1.00–2.00 ml) was collected into a 15-ml sterile falcon tube (Axygen, Tewksbury, MA, USA), and biopsy synovium (~2–3 g) was collected during the surgery into a sterile falcon tube containing 10 ml Dulbecco’s modified Eagle’s medium (DMEM) with 10% fetal bovine serum (FBS). Patients with OA who had TKR (*N* = 47) or UKR (*N* = 13) were included in the study. Plasma samples were separated from whole blood by centrifugation at 1,300 × *g* for 10 min at 4°C, aliquoted, and stored at −80°C until further use. The demographic and clinical parameters are displayed in **Supplementary Table S1**.

### Isobaric tag for relative and absolute quantitation labeling

The plasma samples from OA patients (*N* = 36) and HCs (*N* = 36) were pooled separately, and three groups (each consisting of 12 samples) were prepared and processed for in-solution trypsin digestion using 60 µg protein from each group. The proteins were subjected to buffer exchange with triethylammonium bicarbonate (TEAB) buffer, followed by treatment with 2 mM dithiothreitol (DTT) and incubation at 60°C for 30 min. After incubation, the proteins were treated with 2 mM iodoacetamide (IAA) and incubated for another 30 min at room temperature (RT), followed by trypsin (1:10) digestion (Promega, Madison, WI, USA) at 37°C for 18 h. Digested peptides were labeled with different isobaric tags (OA patients, tag115; HCs, tag117) and incubated for 2 h at RT. The labeled peptides were then loaded in liquid chromatography followed by tandem mass spectrometry (LC-MS/MS) analysis and quantified by iTRAQ for quantitative analysis (27). On those proteins with two or more unique peptides with an unused score ≥2 were considered. The ratios of the intensities of the reporter ions corresponding to OA were compared with those of HCs in order to determine the fold change of the expressed proteins. Proteins with fold change values less than 0.8-fold downregulated or greater than 1.2-fold upregulated, expressed with a significant *p*-value ≤0.05, were considered to be differentially expressed. At least three independent experiments were run.

### Sequential window acquisition of all theoretical mass spectra analysis

Plasma samples (100 µl) from the pooled samples (OA patients = 36, HCs = 36) were subjected to albumin depletion (Millipore, Burlington, MA, USA) so as not to obscure highly abundant plasma proteins. The protein concentration was measured using the bicinchoninic acid (BCA; Pierce, Rockford, IL, USA) method as per protocol (28). A total of 100 µg of protein (from the OA and HC pooled samples) was trypsin digested after treatment with DTT and IAA, as mentioned above. For SWATH-MS data acquisition, a total of eight datasets for each elution peak were collected to calculate 31 precursors, with an isolation window size of 25 Da for the mass range 350–1250 *m*/*z* and a permitted overlap of 1 Da. The MS/MS scan was acquired in the mass range 100–1500 *m*/*z*. The filling time for the MS scan was 250 ms and for the MS/MS scan was 100 ms (29). Proteins with at least two signature unique peptides were considered for the study.

### Protein–protein interaction of the screened proteins related to fibrosis

The proteins that were identified from the MS analysis were matched with the proteins associated with fibrosis retrieved from the DisGeNET database to screen the fibrosis-related differentially regulated proteins. A protein–protein interaction (PPI) network of the screened proteins was constructed using Cytoscape 3.8.2 software for the generation of networks, visualization, and for the analysis of interacting proteins. The network is generally used for the identification of target proteins and to determine the pathways involved in a disease condition (30).

### Gene Ontology and Kyoto Encyclopedia of Genes and Genomes enrichment analysis of selected targets

To interpret the properties and functions of the screened proteins, the associated biological processes, cellular components, molecular functions of the targets, and pathway analysis were elucidated using Gene Ontology **(**GO) and Kyoto Encyclopedia of Genes and Genomes (KEGG) analyses through Cytoscape 3.8.2 and were represented by R studio. The GO analysis tool was used for gene function studies related to their biological processes and molecular functions (31, 32). The KEGG database was used to view genes as perturbed states of the pathway maps and the gene/molecule list of a given dataset.

### Western blot

Plasma samples were pooled as mentioned *Section 2.3*. Briefly, 40 µg of protein from the pooled plasma (*N* = 3) samples (OA patients = 36, HCs = 36) were run in 12% sodium dodecyl sulfate–polyacrylamide gel electrophoresis (SDS-PAGE) by applying 60 V constant voltage. Similarly, the pooled SF samples (TKR = 12, UKR = 12) were also run in 12% SDS-PAGE. The proteins were then transferred onto a nitrocellulose membrane (Millipore, Burlington, MA, USA) using the semi-dry WB technique (Bio-Rad, Hercules, CA, USA) for 50 min at 20 V. The membrane was incubated with 5% bovine serum albumin (BSA) overnight at 4°C, followed by incubation with diluted (1:3,000) anti-LRG1 primary antibody (BioString, Pennsylvania, PA, USA) for 2 h. The blot was then washed with a wash buffer [0.05% Tween-20 in 1× phosphate-buffered saline PBS)] for 5 min, followed by incubation with diluted (1:5,000) anti-rabbit horseradish peroxidase (HRP)-conjugated secondary antibody. After washing the blot, the expressed protein bands were detected using chemiluminescent substrate and analyzed with Chemidoc (Bio-Rad, Hercules, CA, USA) (33).

### Enzyme-linked immunosorbent assay

ELISA was performed using an ELISA kit (Elabscience, Houston, TX, USA) following the manufacturer’s protocol. Briefly, the plasma samples (OA patients = 60, HCs = 40) were incubated at 37°C for 2 h in a pre-coated ELISA plate (provided), followed by washing with a wash buffer. Diluted (1:100) anti-LRG1 primary antibody was added to the wells and then incubated for 1 h at 37°C. Subsequently, biotinylated secondary antibody (1:100) was added and incubated for 30 min at 37°C. The substrate was added after washing as above and the absorbance measured at 462 nm in an ELISA reader (Molecular Devices, San Jose, CA, USA). The pure LRG1 protein was used as a standard to generate the standard curve and to estimate the LRG1 concentration as per instructions in the manual (28).

### Primary cell culture

Biopsy synovium samples from patients with OA were immediately collected in DMEM containing 10% FBS and transferred to the lab in sterile condition at 4°C. The tissue samples were washed with PBS, separated from any cartilage or adipose tissue, chopped into 1-mm pieces, digested with 0.5 mg/ml collagenase (Sigma, St. Louis, MO, USA), and finally incubated at 37°C for 18 h in DMEM with 10% FBS. The digested tissues were then passed through a cell strainer (BD Biosciences, San Jose, CA, USA), the separated cells seeded in a T-75 tissue culture flask, and then incubated in a CO_2_ incubator at 37°C for further use (34, 35).

### *In vitro* knockdown of LRG1

The primary cells generated above were grown up to three passages in a T-75 tissue culture plate. After three passages, the cells were seeded in a six-well tissue culture plate (Nunc, Rochester, NY, USA) in complete media. The cells were grown until 80% confluency, followed by transfection with 50 nM si-LRG1 (Santa Cruz Biotechnology, Santa Cruz, CA, USA) using liposome the “RNAiMax” (4 µl/ml) transfection reagent (Invitrogen, Carlsbad, CA, USA) in complete media (DMEM with 10% FBS) to knock down the expression of LRG1 in the cells. The cells were then grown further in the transfected media for 48 h (36).

### Scratch and migration assay

The primary cells were grown in six-well tissue culture plates up to 80% confluency with complete media. A vertical scratch was drawn with a pipette tip at the center of the flask, and each scratch area was measured before and after treatments. The cells were then transfected with small interfering RNA (siRNA) for LRG1 or with 100 nM recombinant LRG1 (BioString, Pennsylvania, PA, USA) along with the experimental controls (nonspecific siRNA and HC pools). The cells were further grown in media (DMEM with 10% FBS) for another 72 h, followed by the acquisition of bright-field images using a Nikon Eclipse 650 (NIKON, Tokyo, Japan) at ×10 magnification. The images were then analyzed using ImageJ software, and the scar area was measured by selecting the freehand tool application in ImageJ software (37).

### Hydroxyproline assay

The cells were grown in a T-75 tissue culture flask until 80% confluency, transfected with si-LRG1 along with the controls as above, and incubated for a further 120 h to enhance the secretion of the ECM. The level of 4-hydroxy-l-proline (hydroxyproline) was measured using a hydroxyproline assay kit (Elabscience, Houston, TX, USA). The cells were hydrolyzed with 18 N hydrochloric acid (HCL) and then incubated at 100°C for 4 h until they were hydrolyzed completely. The pH was adjusted as per the protocol using a buffer (provided with the kit) and activated charcoal added to adsorb precipitation, followed by centrifugation at 6,000 × *g* for 10 min. Clear supernatant was then used to detect hydroxyproline by mixing with detection reagents (provided with the kit), followed by incubation at 60°C for 15 min. The optical density (OD) was measured and analyzed using an ELISA reader at 550 nm (Molecular Devices San Jose, CA, USA) (38).

### Real-time polymerase chain reaction

Total RNA was isolated (TRIzol method) after treatment of the cells along with the controls in six-well plates (39). First-strand complementary DNA (cDNA) was synthesized with a cDNA synthesis kit (G-Biosciences, St. Louis, MO, USA) using 2 µg of total RNA as per the manufacturer’s protocol. The RNA quality was checked with NanoDrop (Thermo Scientific, Waltham, MA, USA) and the integrity examined by running the RNA in 1% agarose gel. The primers used for interleukin 6 (IL-6), cartilage oligomeric matrix protein (COMP), and LRG1 were as shown below. SyBr Green master mix and template DNA were used to run RT-PCR in LightCycler® 480 (Roche, Mannheim, Germany). The number of amplification cycles was set to 35, and the *C*_t_ value calculated for each gene expression was normalized with the *C*_t_ value of glyceraldehyde-3-phosphate dehydrogenase (GAPDH). Fold change was calculated using the ΔΔ*^C^* ^t^ method (36).

COMP: 5′-AGTCCGCTGTATCAACACCA-3′

5′-AGTTATGTTGCCCGGTCTCA-3′

IL-6: 5′-GGTACATCCTCGACGGCATCT-3′

5′-GTGCCTCTTTGCTGCTTTCAC-3′

LRG1: 5′-GGACACCCTGGTATTGAAAGAAA-3′

5′-TAGCCGTTCTAATTGCAGCGG-3′

Actin: 5′-CATCCGCAAAGACCTGTACG-3′

5′-CCTGCTTGCTGATCCACATC-3′

GAPDH: 5′-GAAGGTGAAGGTCGGAGTC-3′

5′-GAAGATGGTGATGGGATTTC-3′

### Statistical analysis and software used

All the WB images were taken by Chemidoc (Bio-Rad, Hercules, CA, USA), and densitometry analysis of the images was carried out using Image Lab v6 (Bio-Rad, Hercules, CA, USA). Microscopy images were taken at ×10 magnification using Nikon Eclipse 650 (NIKON, Tokyo, Japan), and analysis was carried with ImageJ analysis software. All graphs were generated and the statistical analyses was carried out using GraphPad Prism (version 8.4.686). Data were presented as the mean ± standard deviation (SD). Statistical analysis was performed with the paired Student’s *t*-test to compare the data between two groups, and one-way analysis of variance (ANOVA) was used to compare data among multiple groups. For all datasets, a *p*-value ≤0.05 was considered statistically significant. The obtained *p*-values were represented by asterisks on the graph (*p ≤ 0.05, **p ≤ 0.01, ***p ≤ 0.001, ****p ≤ 0.0001).

## Results

### Upregulation of LRG1 in OA plasma samples

We identified 192 DEPs, of which 38 were identified by iTRAQ and 154 by SWATH (**Supplementary Table S2**). The analysis revealed that 36 proteins were highly differentially expressed (upregulated ≥3-fold and downregulated ≤0.35-fold), with 24 proteins upregulated and 12 proteins downregulated (**Table 1**). Of all the DEPs, LRG1 appeared to be the most highly upregulated (9.46-fold upregulated), while serotransferrin, an iron transporter protein, was the most downregulated (7.6-fold downregulated) in the plasma samples from patients with OA compared to those from HCs.

**Table 1:** Highly differentially expressed proteins (upregulated ≥3-fold and downregulated ≤0.35-fold) identified using an isobaric tag for relative and absolute quantitation (iTRAQ) and sequential window acquisition of all theoretical mass spectra (SWATH-MS).

No.	Name	Fold change
**Highly upregulated differential proteins (≥3-fold)**		
1	Alpha-1-acid glycoprotein 1	4.28E+00
2	Fibrinogen alpha chain	5.44E+00
3	Leucine-rich alpha-2-glycoprotein	9.46E+00
4	Alpha-1-antitrypsin	4.58E+00
5	Vitamin D-binding protein	3.13E+00
6	Apolipoprotein A-II	3.00E+00
7	Histidine-rich glycoprotein	3.66E+00
8	*N*-acetylmuramoyl-l-alanine amidase	5.15E+00
9	Immunoglobulin kappa constant	7.18E+00
10	Angiotensinogen	6.39E+00
11	Heparin cofactor 2	4.41E+00
12	Complement component C8 gamma chain	5.00E+00
13	APOC4–APOC2 readthrough (NMD candidate)	3.36E+00
14	Ficolin-3	3.14E+00
15	Immunoglobulin kappa variable 3-15	6.67E+00
16	Complement factor H-related protein 1	6.36E+00
17	Properdin	4.55E+00
18	Trypsin-1 (fragment)	6.15E+00
19	IgGFc-binding protein (fragment)	3.43E+00
20	Immunoglobulin heavy variable 3-13	3.25E+00
21	Bridging integrator 3	4.09E+00
22	Probable helicase with zinc finger domain	3.81E+00
23	Beta-2-microglobulin (fragment)	9.14E+00
24	Transcription factor Sp5	7.06E+00
**Highly downregulated differential proteins (≤0.35-fold)**		
1	Complement C3	1.59E−01
2	Hemoglobin subunit beta	1.60E−01
3	Apolipoprotein A-IV	3.47E−01
4	Coagulation factor XIII A chain	2.38E−01
5	Serotransferrin	1.31E−01
6	cDNA FLJ55673, highly similar to complement factor B	3.13E−01
7	Prothrombin	1.94E−01
8	Inter-alpha-trypsin inhibitor heavy chain H1	3.55E−01
9	Apolipoprotein C-I (fragment)	2.33E−01
10	Complement C1r subcomponent-like protein	2.71E−01
11	Complement C2	2.68E−01
12	Serum amyloid A protein	2.90E−01

### Validation of LRG1 in plasma and synovial fluid samples

**Figure 1:**
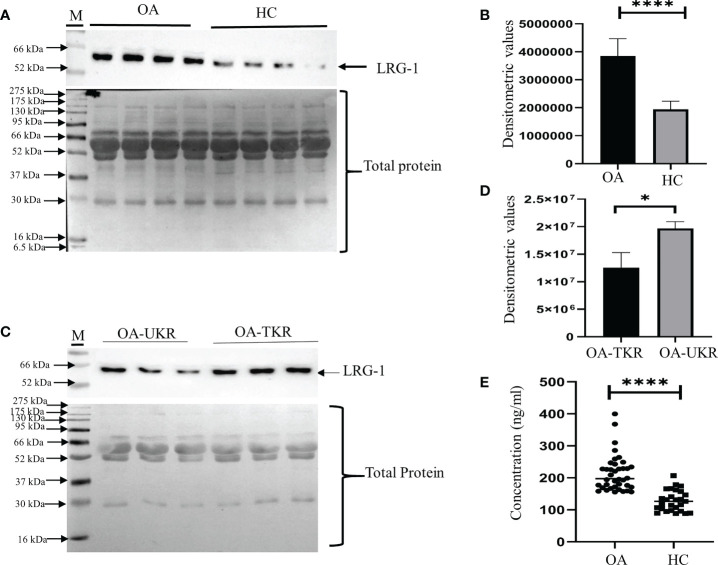
Validation of LRG1 by Western blot (WB). WB images were taken by Chemidoc (Bio-Rad, Hercules, CA, USA), and densitometry analysis of all WB images was carried out using Image Lab v6 (Bio-Rad). All graphs were generated and the statistical analysis carried out using GraphPad Prism (version 8.4.686). Data are presented as the mean ± standard deviation (SD). Paired Student’s *t*-test was performed for group comparisons in **(B, D, E)** to show statistical significance. **(A)** WB image of the expression of LRG1 in pooled plasma samples from patients with OA and from HCs (*upper panel*) and Ponceau staining of blots showing total protein as the loading control (*lower panel*). **(B)** Graph of the densitometric analysis of the WB images using Image Lab software. LRG1 was found to be 2.3-fold upregulated in the plasma samples from OA patients compared to those from HCs. **(C)** WB images of the LRG1 expression and Ponceau staining of blots showing total protein in the SF samples from patients who had TKR compared to those who had UKR. **(D)** Bar graph showing the 1.6-fold upregulation of LRG1 in SF samples (*p* = 0.015). **(E)** ELISA results showing the concentrations of LRG1 in plasma samples from patients with OA compared to those from HCs. The fold change was 1.66 in OA compared to HC plasma. *p ≤ 0.05; ****p ≤ 0.0001. *ns*, non-significant; *LRG1*, leucine-rich alpha-2 glycoprotein; *OA*, osteoarthritis; *SF*, synovial fluid; *HC*, healthy control; *TKR*, total knee replacement; *UKR*, unicompartmental knee replacement.

The upregulation of LRG1 was validated by WB in the pooled (*N* = 12) plasma samples of OA patients (*N* = 36) compared to HCs (*N* = 36). The LRG1 level was found to be 2.3-fold increased in the plasma samples from patients with OA compared to those from HCs (**Figures 1A, B**), which was highly significant (*p* = 0.0001). The upregulation of LRG1 was also validated by ELISA in 60 plasma samples from OA patients (TKR+UKR) and 40 HCs, which revealed a 1.66-fold upregulation in the OA samples compared to HCs (**Figure 1E**). The expression level of LRG1 was further examined in the SF samples from TKR (*N* = 12) and compared with that from UKR (*N* = 12) (**Figure 1C**). The densitometric values of LRG1 revealed a 1.66-fold upregulation of the protein in patients who had TKR compared to those who had UKR (*p* = 0.015) (**Figure 1D**)

### Association of LRG1 with the fibrosis-related proteins using protein–protein interaction network analysis

**Figure 2:**
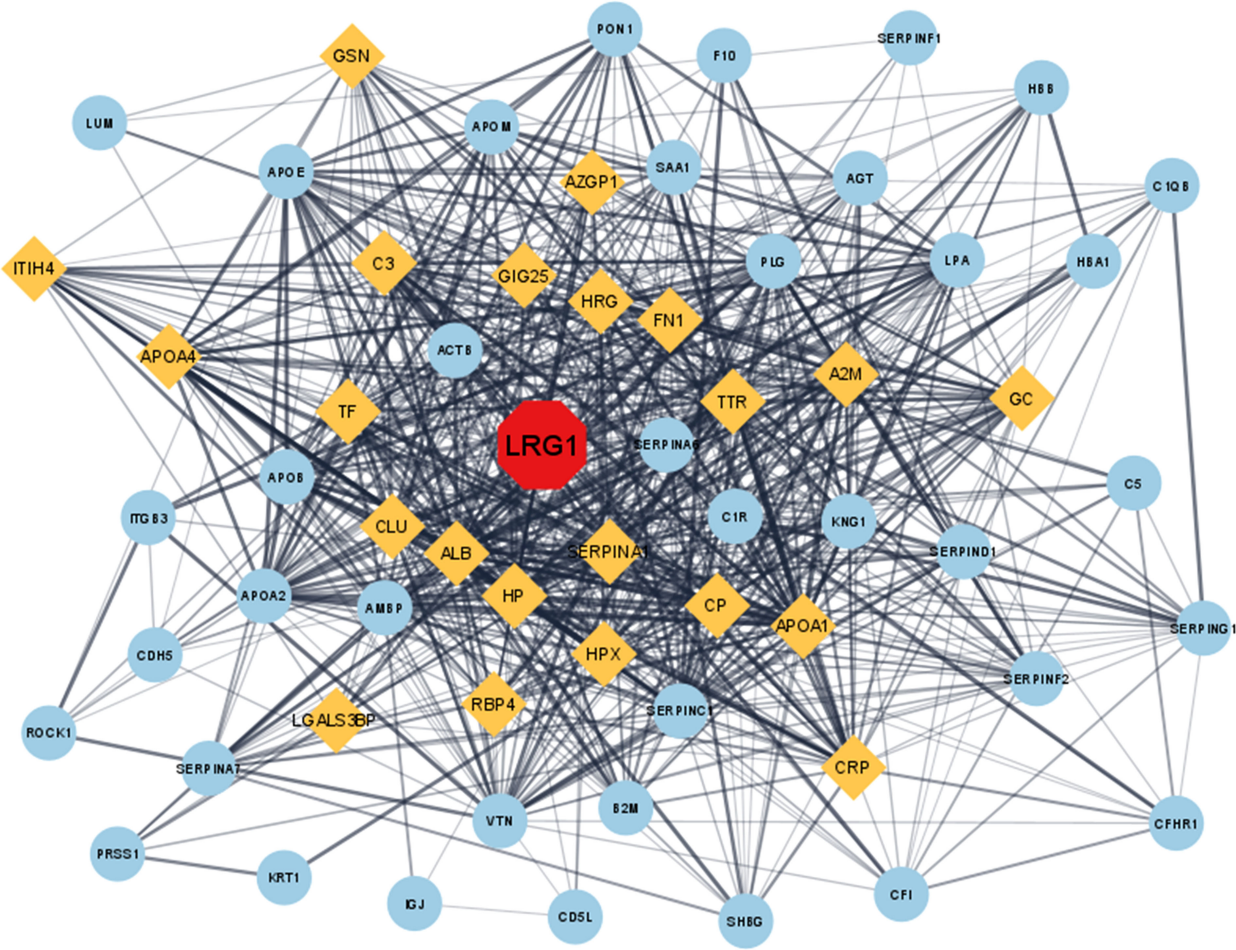
*In silico* study of the DEPs involved in fibrosis-related disease. The proteins were converted to their corresponding gene ID and analyzed using the Cytoscape (3.8.2) online analysis tool. The protein–protein interaction in Cytoscape showed that LRG1 plays a central role in fibrosis (*red*). Closely associated proteins are shown in *yellow*, while the other distantly related proteins are shown as *sky blue*. *LRG1*, leucine-rich alpha-2 glycoprotein; *DEPs*, differentially expressed proteins.

A total of 63 DEPs were screened after matching the identified DEPs in OA from the MS analysis and the fibrosis-related proteins retrieved from the DisGeNET database. The PPI network of the 63 proteins depicted LRG1 as one of the important proteins involved in fibrosis and also visualized its direct and indirect interacting proteins (**Figure 2**). LRG1 was depicted in red color, while the primary neighboring proteins—AZGP1, C3, GIG25, HRG, FN1, TTR, A2M, GSN, CP, SERPINA, APOA1, HPX, HP, ALB, CLU, TF, APOA4, ITIH4, LGALS3BP, RBP4, CRP, and GC—were depicted in orange color (**Figure 2**).

### Association of LRG1 with the fibrosis-related proteins

**Figure 3:**
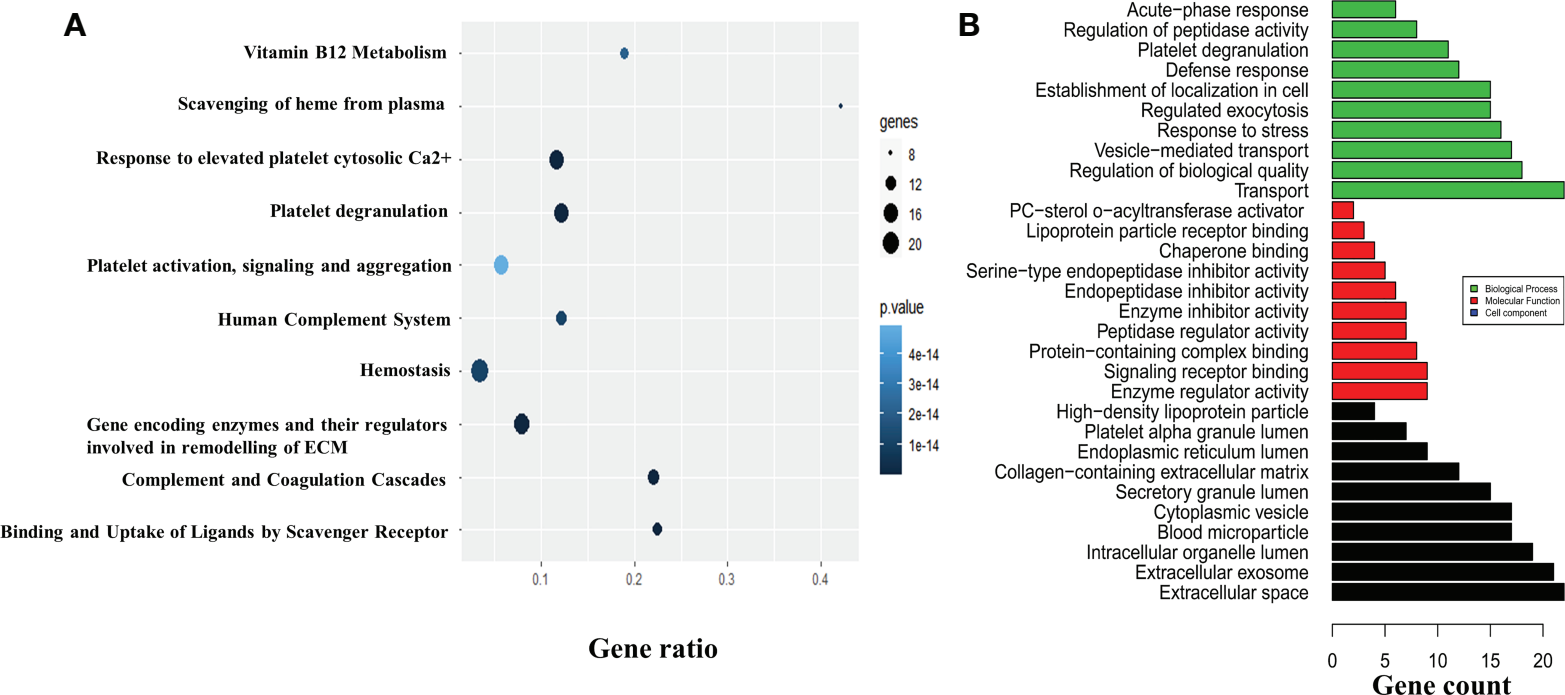
*In silico* study and annotation. **(A)** The DEPs were used to map the pathways involved in the pathogenesis of osteoarthritis (OA). The pathways are depicted by the *colored circle*, where the *size of the circle* indicates the number of proteins and the *color of the circle* indicates the *p*-value. **(B)** The GO annotation tool was used to determine the molecular and functional roles of the identified DEPs. The number of proteins involved in biological processes, molecular functions, and cell components are shown in *green*, *red*, and *black*, respectively. The top 10 GO entries with *p* ≤ 0.05 are depicted. *DEPs*, differentially expressed proteins; *GO*, Gene Ontology; *ECM*, extracellular matrix.

The 63 proteins screened were subjected to functional enrichment analysis in Cytoscape and a list of GO and KEGG enrichment analysis were retrieved respectively. To analyze the relationship between the screened proteins and their biological pathways, a bubble plot was constructed using the KEGG enrichment data, which depicted the highest number of identified proteins involved in the homeostasis and remodeling of the ECM (**Figure 3A**). The GO analysis of these 63 proteins retrieved a total of 134 GO entries, of which 93 entries were associated with biological processes that mainly included transport, response to stress, and establishment of localization in cell; 17 entries depicted molecular functions, which included enzyme regulator activity and signaling receptor binding; and 24 proteins that were related to cell component, mainly extracellular space and exosome (**Figure 3B**). Fibrosis was indicated as one of the major pathways in the analysis of pathways involved in the regulation of the ECM, possibly associated with the development of joint fibrosis in patients with OA. The upregulation of LRG1 and its association with other fibrosis-related proteins indicated that LRG1 might play a central role in fibrosis in OA.

### Knockdown of LRG1 reduces the mRNA levels of the inflammatory and fibrosis markers

**Figure 4:**
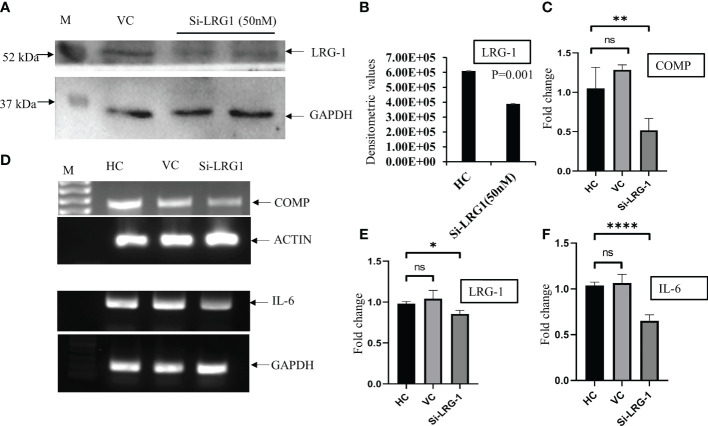
LRG1 promotes fibrosis in primary synovial cells. Western Blot (WB) and agarose gel images were taken by Chemidoc (Bio-Rad, Hercules, CA, USA), and densitometry analysis of all the images was carried out using Image Lab V6 (Bio-Rad). All graphs were generated and the statistical analysis carried out using GraphPad Prism (version 8.4.686). Data are presented as the mean ± standard deviation (SD). Paired Student’s *t*-test was applied for groups comparisons in **(B)** to show statistical significance, while one-way ANOVA was used for group comparisons in **(C, E, F)**. **(A)** Western blot image showing LRG1 knockdown using siRNA (50 nM) in primary fibroblast-like cells isolated from OA synovium. The *upper panel* shows the LRG1 expression level with the transfection reagent for 48 h, while the *lower panel* shows GAPDH as the loading control in the respective sample. **(B)** Bar graph plotted using the densitometric values after the analysis of bands depicting the 60% downregulation of the LRG1 expression after knockdown using siRNA. **(C)** Bar graph showing the mRNA level of COMP after the knockdown of LRG1 in OA primary cells compared to the vehicle control group. **(D)** PCR products run on 1% agarose gel for visualization of the mRNA expression levels of IL-6 and COMP after treatment with siRNA. **(E)** Bar graph showing the mRNA level of LRG1 after knockdown. **(F)** Bar graph showing the mRNA level of the inflammatory marker IL-6 after the knockdown of LRG1 compared to the untreated groups. *p ≤ 0.05; **p ≤ 0.01; ****p ≤ 0.0001. *ns*, non-significant; *LRG1*, leucine-rich alpha-2 glycoprotein; *OA*, osteoarthritis; *COMP*, cartilage oligomeric matrix protein; *IL-6*, interleukin 6; *VC*, vehicle group; *HC*, healthy control; *GAPDG*, glyceraldehyde-3-phosphate dehydrogenase.

An approximately 60% knockdown of the LRG1 protein was achieved, which was validated in the WB using si-LRG1 (**Figures 4A, B**). After the knockdown of LRG1, significantly decreased messenger RNA (mRNA) levels of IL-6, a widely recognized inflammatory marker, and COMP, a widely used fibrosis marker, were observed. The mRNA expression levels of COMP, LRG1, and IL-6 were evaluated using RT-PCR and were found to be downregulated by 0.51, 0.85, and 0.69-fold, respectively, after the knockdown of LRG1 (**Figures 4C, E, F**). The expression levels of IL-6 and COMP in the presence of si-LRG1 were determined by running the PCR product in 1% agarose gel (**Figure 4D**).

### Stimulation by LRG1 enhances fibrosis in primary synovial cells

**Figure 5:**
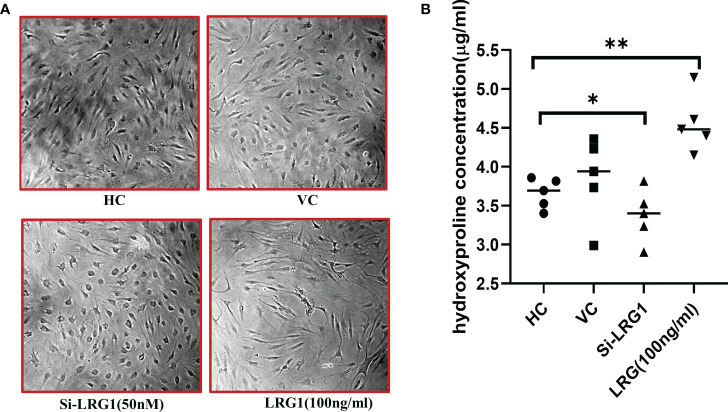
Hydroxyproline assay after stimulation/knockdown of LRG1. Microscopy images were taken at ×10 magnification using Nikon Eclipse 650 (NIKON, Tokyo, Japan). The optical density of hydroxyproline was measured with a spectrophotometer (Molecular Devices, San Jose, CA, USA). Graphs were generated and the statistical analysis carried out using GraphPad Prism (version 8.4.686). Data are presented as the mean ± standard deviation (SD). One-way ANOVA was used to compare the data among groups to show statistical significance. **(A)** Increased extracellular matrix observed in primary synovial cells from patients with OA after stimulation using pure LRG1 (100 ng/ml); a reverse effect was observed morphologically after silencing the LRG1 gene using 50 nM siRNA compared to the non-treated group. **(B)** Level of collagen measured with the hydroxyproline assay. The level of hydroxyproline indicated a 1.3-fold increase in the stimulated (with 100 ng/ml LRG1) group compared to the control group, with 0.74 and 0.9-fold decreased levels of hydroxyproline in the knockdown (50 nm si-LRG1) group compared to the LRG1-treated and the non-treated group, respectively. *p ≤ 0.05; **p ≤ 0.01. *LRG1*, leucine-rich alpha-2 glycoprotein; *OA*, osteoarthritis.

It was observed that the level of hydroxyproline was increased when the cells were stimulated with 100 ng/ml pure recombinant LRG1 (BioString, Pennsylvania, PA, USA) compared to untreated/control cells (**Figure 5A**). The results showed that the level of hydroxyproline significantly decreased by 0.74-fold in the si-LRG1-transfected group (**Figure 5B**) compared to the LRG1-treated group (100 ng/ml) and by 0.9-fold compared to HCs, indicating that LRG1 enhanced the secretion of the ECM on primary synovial cells. Increased ECM was observed in the primary synovial cells of patients with OA after stimulation with LRG1 using pure LRG1 (100 ng/ml), but the reverse effect was observed morphologically after silencing the LRG1 gene using 50 nM siRNA compared to the non-treated group. The level of hydroxyproline is widely known for its association with collagen content and, hence, is used as a marker for the severity of fibrosis (36). Our results indicated that the increased level of LRG1 correlated with an increased level of hydroxyproline, which may therefore lead to synovial fibrosis, thus strengthening our finding.

### LRG1 promotes wound healing and migration in primary synovial cells

**Figure 6:**
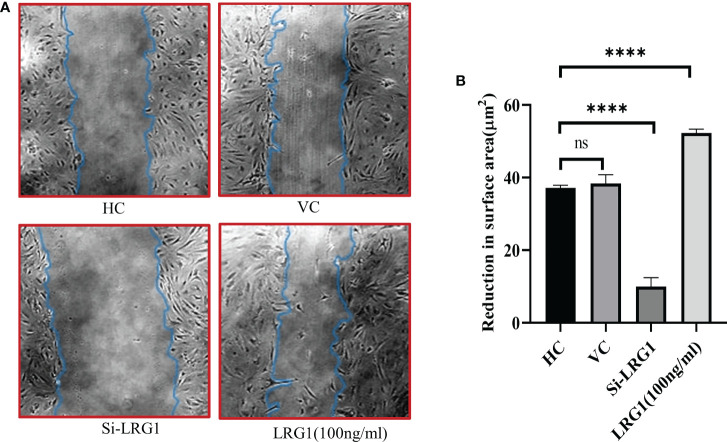
Wound healing and scratch assay. Microscopy images taken at ×10 magnification using Nikon Eclipse 650 (NIKON, Tokyo, Japan). Analysis was carried out with ImageJ software using the freehand area selection tool. Graphs were generated and the statistical analysis carried out using GraphPad Prism (version 8.4.686). Data are presented as the mean ± standard deviation (SD). One-way ANOVA was used for comparisons of the data among groups to show statistical significance. **(A)** Wound healing and migration of primary synovial cells after stimulation with pure LRG1 (100 ng/ml) or knockdown with si-LRG1 (50 nM). **(B)** Graph showing wound healing of OA primary cells after knockdown with siRNA (50 nM) or stimulation with recombinant LRG1 as a function of reduction in the scratch surface area after 72 h of treatment. The results showed a 0.026-fold reduction in the wound healing capacity after knockdown, but a reverse effect was seen when the cells were stimulated with pure LRG1, which showed a 1.4-fold increased wound healing and migration compared to untreated cells. ****p ≤ 0.0001. *ns*, non-significant; *LRG1*, leucine-rich alpha-2 glycoprotein; *OA*, osteoarthritis.

In order to determine the effect of LRG1 on the migration of synovial cells, a wound-healing assay was performed after the knockdown of LRG1. The results indicated a decrease in the migration of cells in the group transfected with si-LRG1 compared to the HC and the vehicle control group (transfected with nonspecific siRNA pool). Interestingly, the group treated with 100 ng/ml of pure LRG1 showed an enhancement in the migration and healing process compared to the group transfected with siRNA and to the HCs (**Figure 6A**). The reduction in the scratch surface area in the siRNA-treated group was found to be 0.26-fold lower than that in HCs, which was significant (*p* ≤ 0.0001), while this effect was reversed upon treatment with 100 nM recombinant LRG1, which showed a 1.4-fold increase in the scratch surface area compared to that in HCs (*p* = 0.0001) (**Figure 6B**).

## Discussion

OA, a common joint disease characterized by deformity and pain, leads to restricted synovial joint movement due to synovial joint stiffness, primarily caused by joint fibrosis (40). Reports have shown that most patients with OA develop symptoms of synovial fibrosis; however, it is broadly ignored during diagnosis and treatment (3, 9). Since fibrosis is the factor responsible for joint stiffness that leads to pain and restriction in movements (7), we speculated that an intervention targeting the fibrosis cascade might help in the better management of this disease and improve the treatment approaches.

To understand the pathological mechanism behind joint fibrosis, we screened 63 fibrosis-related differential proteins, which identified LRG1 as the most highly upregulated (9.4-fold) protein (**Table 1**). LRG1 has also been studied earlier in liver and kidney fibrosis (22, 41), causing pathogenesis in fibrosis-related diseases such as ocular and skin fibrosis (22), which indicates that LRGI is a potential candidate biomarker in joint fibrosis. Our bioinformatics studies (pathway analysis using PPI and GO) also demonstrated the involvement of LRG1 in joint fibrosis, and its associated proteins were found to be linked with modifications in the ECM (**Figure 3A**). Further *in silico* studies revealed that the acute phase inflammatory proteins transthyretin (TTR), complement-3 (C3), inter-alpha-trypsin inhibitor heavy chain 4 (ITIH4), C-reactive protein (CRP), and haptoglobin (HP) are closely associated with LRG1 (**Figure 2A**). The level of LRG1 was also found to be correlated with CRP, erythrocyte sedimentation rate (ESR), and disease activity score 28 (DAS28), but not with the serum TNF-α levels in rheumatoid arthritis (RA) (42). Here, it should be noted that inflammation of the joints remains the common factor affecting individuals with RA and OA; moreover, the DAS28 score is based on the number of joints affected by the disease, indicating the association of LRG1 with joint health. TTR, ITIH4, C3, and CRP have been previously reported in the disease pathogenicity and inflammation of autoimmune diseases, affecting synovial joints with restricted joint movements, whereas HP has been linked with irregularity in the homeostasis of free hemoglobin in OA (43). Furthermore, the close association of LRG1 (based on our *in silico* studies) with the pro-inflammatory proteins (i.e., TTR, ITIH4, C3, and CRP) suggests that it is associated with joint fibrosis and inflammation in OA. Moreover, a higher level of LRG1 (1.6-fold) was observed in the SF samples of patients who had TKR compared to those who underwent UKR (**Figure 1C**), which may be due to the degree of joint freedom being lower in TKR than in UKR. As both cruciate ligaments and the two knee compartments are preserved in patients who had UKR, they have normal gait and better knee flexion compared to those who underwent TKR (13). Moreover, the level of LRG1 was also correlated with the level of hydroxyproline. An increased level of LRG1 in the plasma samples from patients who had TKR compared to those who had UKR was also observed (**Supplementary Figure S1**), indicating that LRG1 is associated with joint fibrosis in patients with OA. Therefore, we can assume that the level of joint stiffness is a primary factor affecting the level of LRG1 in patients who had TKR compared to those who had UKR. To further validate our finding, an *in vitro* study was carried out using primary synovial cells generated from the biopsy synovium of patients with OA, which investigated the effect of LRG1 on the factors associated with fibrosis, such as wound healing and cell migration. We observed that the stimulation of primary cells with pure LRG1 protein (100 nM) led to a potential wound healing capability and excessive secretion of the ECM in the treated group compared to the non-treated group (**Figure 5**). On the contrary, when the level of LRG1 was knocked down with siRNA, a significant reverse effect was observed, measured as a significantly reduced (0.74-fold) hydroxyproline level (**Figure 5**). The level of hydroxyproline was found to be correlated with the collagen content of the ECM, which can be used to evaluate tissue fibrosis (22, 36, 41). We also observed that the knockdown of LRG1 was associated with a decreased cell migration and a slower wound healing process in primary OA synovial cells, while cells stimulated with LRG1 showed rapid migration and wound healing (**Figure 6**). Reports from the literature also suggested several other factors, including TGF-β, that contribute to the secretion of the ECM (40), which are found in various fibrosis-related diseases. However, our data suggest that the upregulation of LRG1 is one of the primary factors promoting ECM synthesis, which may lead to synovial joint stiffness in OA. Serotransferrin, another significant differential protein, was found to be the most downregulated (**Table 1**), which has a role in iron transport (44) and affects the “homeostasis” pathway, as suggested by GO analysis (**Figure 3A**). Studies have also suggested that poor clearance of free hemoglobin is involved in the pathophysiology of OA (28), suggesting that fibrosis might be linked with the iron metabolism of patients with OA, which needs to be evaluated further. It will be interesting to investigate whether the inhibition of LRG1 would mitigate the joint fibrosis-related symptoms, thus helping to maintain better gait and improving the lifestyle of patients with OA. Although a relevant connection of the DEPs with joint fibrosis in patients with OA can be established, the underlying mechanism triggering the increased level of LRG1 in these patients still needs further exploration.

## Conclusion

The upregulation of LRG1 was found to be associated with the extent of joint fibrosis in patients with OA, and its knockdown led to the reduction of fibrosis and the inflammation-related markers in the *in vitro* studies using primary cells from patients with OA. This study, which showed that the upregulation of LRG1 promotes fibrosis, fills the knowledge gap on the relationship between fibrosis, inflammation, and the level of LRG1 in patients with OA.

## Data availability statement

The original contributions presented in the study are included in the article/**Supplementary Material**. Further inquiries can be directed to the corresponding author.

## Ethics statement

The study protocol was approved by the medical Ethics Committee of All India Institute of Medical Sciences (AIIMS), Department of Orthopaedics, New Delhi, India (AIIMS, Ref No CSIR379 IGIB/IHEC2017-18 Dt.08.02.2018) and by the Council of Scientific and Industrial Research (CSIR)-Institute of Genomics and Integrative Biology, Delhi, India, (CSIR IGIB/IHEC/2017-18 Dated 08.02.2018). All study protocol is complied with the Declaration of Helsinki. Written signed consents were obtained from all OA participating patients and healthy volunteers (HC).

## Author contributions

All authors made substantial contributions to the conception and design, acquisition of data, or analysis and interpretation of data; took part in drafting the article or revising it critically for important intellectual content; agreed to submit the current journal; gave final approval of the version to be published; and agreed to be accountable for all aspects of the work.
