# Supplementary material for: Upregulation of leucine-rich alpha-2 glycoprotein: A key regulator of inflammation and joint fibrosis in patients with severe knee osteoarthritis
Source: Front Immunol. 2022 Dec 7;13:1028994. doi: 10.3389/fimmu.2022.1028994 (PMC9768428; doi:10.3389/fimmu.2022.1028994)
Supplement: Supplementary file 1 [file DataSheet_1.docx]

**SUPPLEMENTARY DATA**

**Supplementary Figure 1**


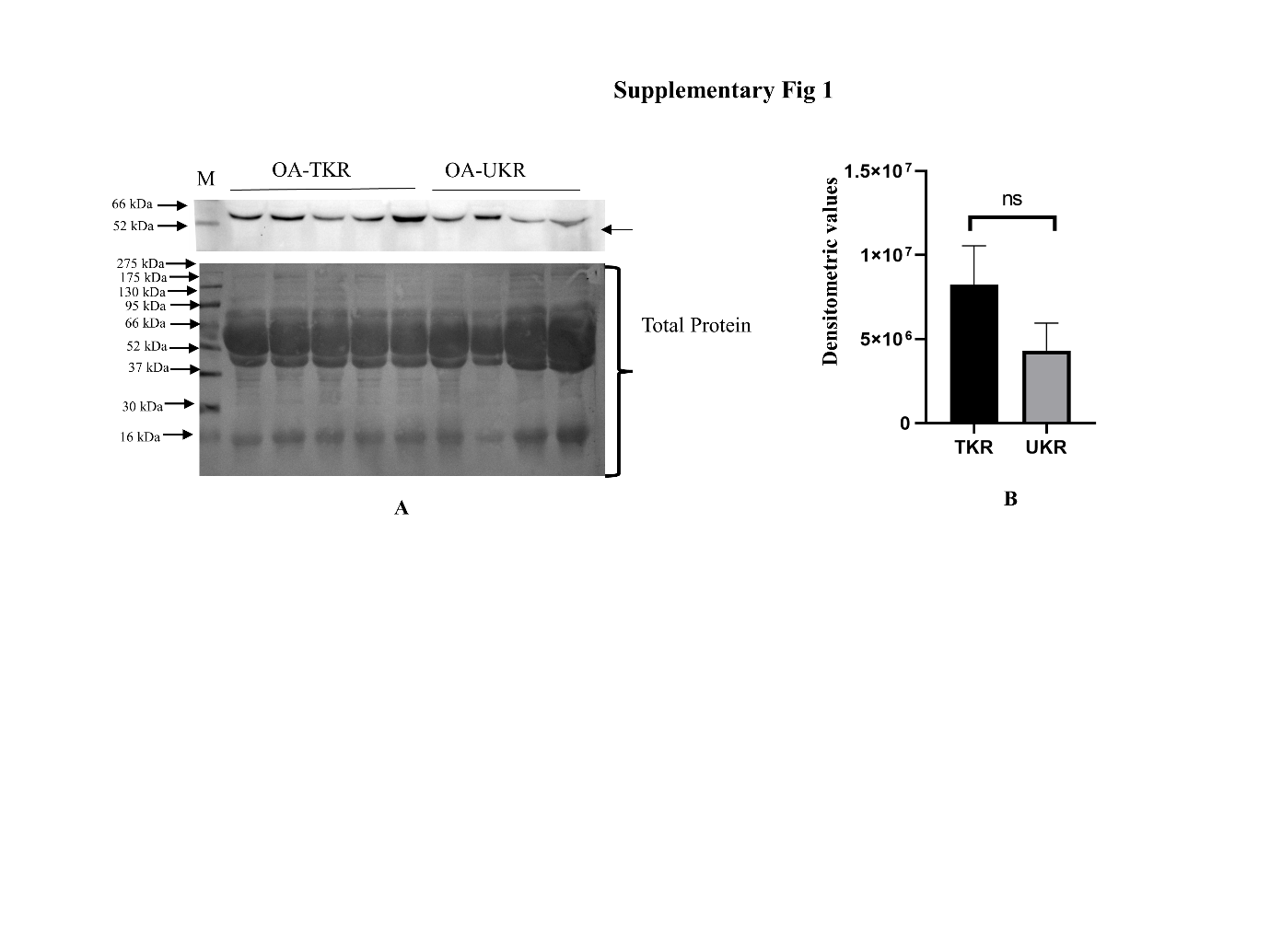
**Supplemtary fig#1: LRG1 level in plasma of TKR/UKR patients. (A)**Western blot image LRG1 and total protein in TKT and UKR plasma sample showing total protein (lower panel) and LRG1 expression (upper panel). **(B)** Bar graph representing level of LRG1 in TKR and UKR plasma sample where LRG1 level has been found non-significantly up regulated in TKR patients compared to UKR patients’ plasma sample. (ns= non-significant, TKR = Total Knee Replacement, UKR =).

B

**Supplementary Table 1**

| **Supplementary Table 1: Clinical measures of patients (TKR=45, UKR=12) and 40 Healthy Control(HC)** | | | | |
| --- | --- | --- | --- | --- |
| **S. No** | characteristics | TKR(n=47) | UKR(n=13) | HC(n=40) |
| **1** | Mean Age(in Years) ±SD | 55 ± 10 | 55 ± 10 | 50± 10 |
| **2** | BMI ±SD | 31±2 | 30±3 | 29±4 |
| **3** | Female (%) | 62% | 66.6% | 68% |
| **4** | Mean ESR ±SD | 20 ± 5 | 10 ± 5 | - |
| **5** | RF | -ve | ­ ve | - |
| **6** | Mean swollen Joint ±SD | 1.5± 0.5 | 1± 0.5 | - |
| **7** | Mean health score(Best =0, worst=100) ± SD | 30±5 | 15 ± 5 | 0 |
| **8** | Mean disease duration(years) ±SD | 7 ± 3 | 4 ±2 | - |
| **9** | Medication(Yes/No) | Yes | Yes | No |

**Supplementary Table 2**

**Table 2: List of differentially expressed proteins identified by Isobaric Tag for Relative and Absolute Quantitation (iTRAQ) and Sequential Window Acquisition of All Theoretical Mass Spectra (SWATH-MS) technique.**

| S No | PROTEIN NAME | FOLD CHANGE |
| --- | --- | --- |
| 1 | Serum albumin OS=Homo sapiens OX=9606 GN=ALB PE=1 SV=2 | 1.1272 |
| 2 | Serotransferrin OS=Homo sapiens OX=9606 GN=TF PE=1 SV=3 | 0.5702 |
| 3 | Alpha-2-macroglobulin OS=Homo sapiens OX=9606 GN=A2M PE=1 SV=3 | 2.5119 |
| 4 | Alpha-1-antitrypsin OS=Homo sapiens OX=9606 GN=SERPINA1 PE=1 SV=3 | 2.9923 |
| 5 | Haptoglobin (Fragment) OS=Homo sapiens OX=9606 GN=HP PE=1 SV=1 | 2.3988 |
| 6 | Hemoglobin subunit alpha OS=Homo sapiens OX=9606 GN=HBA1 PE=1 SV=2 | 4.2073 |
| 7 | Ceruloplasmin OS=Homo sapiens OX=9606 GN=CP PE=1 SV=1 | 2.6792 |
| 8 | Complement C3 OS=Homo sapiens OX=9606 GN=C3 PE=1 SV=2 | 0.1585 |
| 9 | Hemoglobin subunit beta OS=Homo sapiens OX=9606 GN=HBB PE=1 SV=2 | 0.9727 |
| 10 | Apolipoprotein A-I OS=Homo sapiens OX=9606 GN=APOA1 PE=1 SV=1 | 1.0965 |
| 11 | Hemopexin OS=Homo sapiens OX=9606 GN=HPX PE=1 SV=2 | 1.2589 |
| 12 | Alpha-1B-glycoprotein OS=Homo sapiens OX=9606 GN=A1BG PE=1 SV=4 | 1.1695 |
| 13 | Hemoglobin subunit delta OS=Homo sapiens OX=9606 GN=HBD PE=1 SV=2 | 1.1376 |
| 14 | Transthyretin OS=Homo sapiens OX=9606 GN=TTR PE=1 SV=1 | 1.2474 |
| 15 | Haptoglobin-related protein OS=Homo sapiens OX=9606 GN=HPR PE=2 SV=2 | 0.4966 |
| 16 | Alpha-1-acid glycoprotein 1 OS=Homo sapiens OX=9606 GN=ORM1 PE=1 SV=1 | 0.5445 |
| 17 | Alpha-2-HS-glycoprotein OS=Homo sapiens OX=9606 GN=AHSG PE=1 SV=1 | 1.5996 |
| 18 | Vitamin D-binding protein OS=Homo sapiens OX=9606 GN=GC PE=1 SV=1 | 1.0864 |
| 19 | Immunoglobulin heavy constant alpha 1 (Fragment) OS=Homo sapiens OX=9606 GN=IGHA1 PE=1 SV=1 | 1.1912 |
| 20 | Apolipoprotein A-IV OS=Homo sapiens OX=9606 GN=APOA4 PE=1 SV=3 | 0.8395 |
| 21 | Alpha-2-antiplasmin OS=Homo sapiens OX=9606 GN=SERPINF2 PE=1 SV=1 | 1.6444 |
| 22 | Afamin OS=Homo sapiens OX=9606 GN=AFM PE=1 SV=1 | 0.8241 |
| 23 | Alpha-1-acid glycoprotein 2 OS=Homo sapiens OX=9606 GN=ORM2 PE=1 SV=2 | 1.5276 |
| 24 | Apolipoprotein A-II OS=Homo sapiens OX=9606 GN=APOA2 PE=1 SV=1 | 1.6444 |
| 25 | ITIH4 protein OS=Homo sapiens OX=9606 GN=ITIH4 PE=1 SV=1 | 0.6607 |
| 26 | Angiotensinogen OS=Homo sapiens OX=9606 GN=AGT PE=1 SV=1 | 1.6904 |
| 27 | Immunoglobulin heavy constant gamma 2 (Fragment) OS=Homo sapiens OX=9606 GN=IGHG2 PE=1 SV=1 | 0.6138 |
| 28 | Immunoglobulin heavy constant mu (Fragment) OS=Homo sapiens OX=9606 GN=IGHM PE=1 SV=1 | 0.6486 |
| 29 | Fibrinogen alpha chain OS=Homo sapiens OX=9606 GN=FGA PE=1 SV=2 | 5.445 |
| 30 | Leucine-rich alpha-2-glycoprotein OS=Homo sapiens OX=9606 GN=LRG1 PE=1 SV=2 | 9.4624 |
| 31 | Coagulation factor XIII A chain OS=Homo sapiens OX=9606 GN=F13A1 PE=1 SV=4 | 0.2377 |
| 32 | Serum paraoxonase/arylesterase 1 OS=Homo sapiens OX=9606 GN=PON1 PE=1 SV=3 | 2.5351 |
| 33 | Antithrombin-III OS=Homo sapiens OX=9606 GN=SERPINC1 PE=1 SV=1 | 0.5058 |
| 34 | Carbonic anhydrase 1 OS=Homo sapiens OX=9606 GN=CA1 PE=1 SV=2 | 2.421 |
| 35 | Apolipoprotein B-100 OS=Homo sapiens OX=9606 GN=APOB PE=1 SV=2 | 1.5417 |
| 36 | Carboxypeptidase N subunit 2 OS=Homo sapiens OX=9606 GN=CPN2 PE=1 SV=3 | 1.7539 |
| 37 | Protein AMBP OS=Homo sapiens OX=9606 GN=AMBP PE=1 SV=1 | 0.8091 |
| 38 | Serum albumin OS=Homo sapiens OX=9606 GN=ALB PE=1 SV=2 | 2.06064 |
| 39 | Apolipoprotein B-100 OS=Homo sapiens OX=9606 GN=APOB PE=1 SV=2 | 0.31232 |
| 40 | Complement C3 OS=Homo sapiens OX=9606 GN=C3 PE=1 SV=2 | 0.22023 |
| 41 | Alpha-2-macroglobulin OS=Homo sapiens OX=9606 GN=A2M PE=1 SV=3 | 0.78696 |
| 42 | Serotransferrin OS=Homo sapiens OX=9606 GN=TF PE=1 SV=3 | 0.30112 |
| 43 | Complement C4-A OS=Homo sapiens OX=9606 GN=C4A PE=1 SV=2 | 0.42155 |
| 44 | Ceruloplasmin OS=Homo sapiens OX=9606 GN=CP PE=1 SV=1 | 0.30805 |
| 45 | Alpha-1-antitrypsin OS=Homo sapiens OX=9606 GN=SERPINA1 PE=1 SV=3 | 1.89692 |
| 46 | Immunoglobulin heavy constant gamma 1 (Fragment) OS=Homo sapiens OX=9606 GN=IGHG1 PE=1 SV=1 | 0.38266 |
| 47 | Fibronectin OS=Homo sapiens OX=9606 GN=FN1 PE=1 SV=4 | 0.30647 |
| 48 | Fibrinogen alpha chain OS=Homo sapiens OX=9606 GN=FGA PE=1 SV=2 | 1.19292 |
| 49 | Complement factor H OS=Homo sapiens OX=9606 GN=CFH PE=1 SV=4 | 65.8751 |
| 50 | Fibrinogen beta chain OS=Homo sapiens OX=9606 GN=FGB PE=1 SV=2 | 0.67831 |
| 51 | Haptoglobin OS=Homo sapiens OX=9606 GN=HP PE=1 SV=1 | 0.97862 |
| 52 | cDNA FLJ55673, highly similar to Complement factor B OS=Homo sapiens OX=9606 PE=1 SV=1 | 0.7386 |
| 53 | Fibrinogen gamma chain OS=Homo sapiens OX=9606 GN=FGG PE=1 SV=3 | 0.16915 |
| 54 | Plasminogen OS=Homo sapiens OX=9606 GN=PLG PE=1 SV=2 | 0.24313 |
| 55 | ITIH4 protein OS=Homo sapiens OX=9606 GN=ITIH4 PE=1 SV=1 | 1.07884 |
| 56 | Vitamin D-binding protein OS=Homo sapiens OX=9606 GN=GC PE=1 SV=2 | 0.49818 |
| 57 | Apolipoprotein A-I OS=Homo sapiens OX=9606 GN=APOA1 PE=1 SV=1 | 1.35354 |
| 58 | Complement C5 OS=Homo sapiens OX=9606 GN=C5 PE=1 SV=4 | 1.15326 |
| 59 | Prothrombin OS=Homo sapiens OX=9606 GN=F2 PE=1 SV=2 | 0.96503 |
| 60 | Inter-alpha-trypsin inhibitor heavy chain H2 OS=Homo sapiens OX=9606 GN=ITIH2 PE=1 SV=1 | 2.03154 |
| 61 | Immunoglobulin heavy constant mu OS=Homo sapiens OX=9606 GN=IGHM PE=1 SV=4 | 1.04163 |
| 62 | Hemopexin OS=Homo sapiens OX=9606 GN=HPX PE=1 SV=2 | 0.42258 |
| 63 | Apolipoprotein A-IV OS=Homo sapiens OX=9606 GN=APOA4 PE=1 SV=3 | 0.17179 |
| 64 | C4b-binding protein alpha chain OS=Homo sapiens OX=9606 GN=C4BPA PE=1 SV=2 | 0.73104 |
| 65 | Antithrombin-III OS=Homo sapiens OX=9606 GN=SERPINC1 PE=1 SV=1 | 0.2875 |
| 66 | Alpha-1-antichymotrypsin OS=Homo sapiens OX=9606 GN=SERPINA3 PE=1 SV=2 | 0.40232 |
| 67 | Hemoglobin subunit beta OS=Homo sapiens OX=9606 GN=HBB PE=1 SV=2 | 1.30297 |
| 68 | Inter-alpha-trypsin inhibitor heavy chain H1 OS=Homo sapiens OX=9606 GN=ITIH1 PE=1 SV=3 | 0.35591 |
| 69 | Complement component C7 OS=Homo sapiens OX=9606 GN=C7 PE=1 SV=2 | 1.63568 |
| 70 | Gelsolin OS=Homo sapiens OX=9606 GN=GSN PE=1 SV=1 | 0.19149 |
| 71 | Alpha-2-HS-glycoprotein OS=Homo sapiens OX=9606 GN=AHSG PE=1 SV=2 | 0.02919 |
| 72 | Kininogen-1 OS=Homo sapiens OX=9606 GN=KNG1 PE=1 SV=2 | 0.23479 |
| 73 | Alpha-1B-glycoprotein OS=Homo sapiens OX=9606 GN=A1BG PE=1 SV=4 | 0.996 |
| 74 | Beta-2-glycoprotein 1 OS=Homo sapiens OX=9606 GN=APOH PE=1 SV=3 | 0.54087 |
| 75 | Apolipoprotein E OS=Homo sapiens OX=9606 GN=APOE PE=1 SV=1 | 0.26617 |
| 76 | Plasma protease C1 inhibitor OS=Homo sapiens OX=9606 GN=SERPING1 PE=1 SV=2 | 0.74853 |
| 77 | Afamin OS=Homo sapiens OX=9606 GN=AFM PE=1 SV=1 | 1.8397 |
| 78 | Complement component C6 OS=Homo sapiens OX=9606 GN=C6 PE=1 SV=3 | 0.60826 |
| 79 | Clusterin OS=Homo sapiens OX=9606 GN=CLU PE=1 SV=1 | 1.03854 |
| 80 | Immunoglobulin lambda-like polypeptide 5 OS=Homo sapiens OX=9606 GN=IGLL5 PE=1 SV=1 | 0.77865 |
| 81 | Alpha-1-acid glycoprotein 1 OS=Homo sapiens OX=9606 GN=ORM1 PE=1 SV=1 | 0.73448 |
| 82 | Apolipoprotein A-II OS=Homo sapiens OX=9606 GN=APOA2 PE=1 SV=1 | 1.76701 |
| 83 | Attractin OS=Homo sapiens OX=9606 GN=ATRN PE=1 SV=2 | 0.24282 |
| 84 | Zinc-alpha-2-glycoprotein OS=Homo sapiens OX=9606 GN=AZGP1 PE=1 SV=2 | 3.78846 |
| 85 | Histidine-rich glycoprotein OS=Homo sapiens OX=9606 GN=HRG PE=1 SV=1 | 1.33354 |
| 86 | Protein AMBP OS=Homo sapiens OX=9606 GN=AMBP PE=1 SV=1 | 0.46952 |
| 87 | N-acetylmuramoyl-L-alanine amidase OS=Homo sapiens OX=9606 GN=PGLYRP2 PE=1 SV=1 | 1.53765 |
| 88 | Immunoglobulin kappa constant OS=Homo sapiens OX=9606 GN=IGKC PE=1 SV=2 | 0.37804 |
| 89 | Alpha-2-antiplasmin OS=Homo sapiens OX=9606 GN=SERPINF2 PE=1 SV=3 | 0.34776 |
| 90 | Pregnancy zone protein OS=Homo sapiens OX=9606 GN=PZP PE=1 SV=4 | 0.52546 |
| 91 | Immunoglobulin heavy constant gamma 2 (Fragment) OS=Homo sapiens OX=9606 GN=IGHG2 PE=1 SV=1 | 0.2812 |
| 92 | Complement C1r subcomponent OS=Homo sapiens OX=9606 GN=C1R PE=1 SV=1 | 3.08688 |
| 93 | Complement C1s subcomponent OS=Homo sapiens OX=9606 GN=C1S PE=1 SV=1 | 0.39401 |
| 94 | Complement component C9 OS=Homo sapiens OX=9606 GN=C9 PE=1 SV=2 | 0.79028 |
| 95 | Angiotensinogen OS=Homo sapiens OX=9606 GN=AGT PE=1 SV=1 | 0.71004 |
| 96 | Transthyretin OS=Homo sapiens OX=9606 GN=TTR PE=1 SV=1 | 0.09412 |
| 97 | Complement factor I OS=Homo sapiens OX=9606 GN=CFI PE=1 SV=1 | 0.60079 |
| 98 | Leucine-rich alpha-2-glycoprotein OS=Homo sapiens OX=9606 GN=LRG1 PE=1 SV=2 | 0.11767 |
| 99 | Actin, cytoplasmic 1 OS=Homo sapiens OX=9606 GN=ACTB PE=1 SV=1 | 0.46155 |
| 100 | Immunoglobulin heavy constant delta OS=Homo sapiens OX=9606 GN=IGHD PE=1 SV=3 | 0.21015 |
| 101 | CD5 antigen-like OS=Homo sapiens OX=9606 GN=CD5L PE=1 SV=1 | 1.79527 |
| 102 | Inter-alpha-trypsin inhibitor heavy chain H3 OS=Homo sapiens OX=9606 GN=ITIH3 PE=1 SV=1 | 0.65512 |
| 103 | Serum paraoxonase/arylesterase 1 OS=Homo sapiens OX=9606 GN=PON1 PE=1 SV=3 | 1.48463 |
| 104 | Vitronectin OS=Homo sapiens OX=9606 GN=VTN PE=1 SV=1 | 0.14069 |
| 105 | Galectin-3-binding protein OS=Homo sapiens OX=9606 GN=LGALS3BP PE=1 SV=1 | 1.552 |
| 106 | Pigment epithelium-derived factor OS=Homo sapiens OX=9606 GN=SERPINF1 PE=1 SV=4 | 0.49816 |
| 107 | Corticosteroid-binding globulin OS=Homo sapiens OX=9606 GN=SERPINA6 PE=1 SV=1 | 0.35634 |
| 108 | Plasma kallikrein (Fragment) OS=Homo sapiens OX=9606 GN=KLKB1 PE=1 SV=1 | 0.28795 |
| 109 | Thyroxine-binding globulin OS=Homo sapiens OX=9606 GN=SERPINA7 PE=1 SV=2 | 0.46321 |
| 110 | Lumican OS=Homo sapiens OX=9606 GN=LUM PE=1 SV=2 | 0.59325 |
| 111 | Retinol-binding protein OS=Homo sapiens OX=9606 GN=RBP4 PE=1 SV=2 | 0.72291 |
| 112 | Carboxypeptidase N subunit 2 OS=Homo sapiens OX=9606 GN=CPN2 PE=1 SV=3 | 0.74217 |
| 113 | Apolipoprotein D (Fragment) OS=Homo sapiens OX=9606 GN=APOD PE=1 SV=1 | 0.37474 |
| 114 | Insulin-like growth factor-binding protein complex acid labile subunit OS=Homo sapiens OX=9606 GN=IGFALS PE=1 SV=1 | 0.6109 |
| 115 | Heparin cofactor 2 OS=Homo sapiens OX=9606 GN=SERPIND1 PE=1 SV=3 | 1.76584 |
| 116 | Immunoglobulin heavy constant gamma 3 (Fragment) OS=Homo sapiens OX=9606 GN=IGHG3 PE=1 SV=1 | 0.22584 |
| 117 | Phosphatidylinositol-glycan-specific phospholipase D OS=Homo sapiens OX=9606 GN=GPLD1 PE=1 SV=3 | 0.69761 |
| 118 | Beta-Ala-His dipeptidase OS=Homo sapiens OX=9606 GN=CNDP1 PE=1 SV=2 | 0.8736 |
| 119 | Alpha-1-acid glycoprotein 2 OS=Homo sapiens OX=9606 GN=ORM2 PE=1 SV=2 | 0.26103 |
| 120 | Apolipoprotein C-III OS=Homo sapiens OX=9606 GN=APOC3 PE=1 SV=1 | 0.53393 |
| 121 | Complement component C8 beta chain OS=Homo sapiens OX=9606 GN=C8B PE=1 SV=1 | 0.54178 |
| 122 | Hemoglobin subunit alpha OS=Homo sapiens OX=9606 GN=HBA1 PE=1 SV=2 | 0.73132 |
| 123 | Complement component C8 gamma chain OS=Homo sapiens OX=9606 GN=C8G PE=1 SV=3 | 0.13691 |
| 124 | Coagulation factor XIII B chain OS=Homo sapiens OX=9606 GN=F13B PE=1 SV=3 | 0.33591 |
| 125 | Immunoglobulin J chain OS=Homo sapiens OX=9606 GN=JCHAIN PE=1 SV=4 | 1.2205 |
| 126 | Tetranectin OS=Homo sapiens OX=9606 GN=CLEC3B PE=1 SV=1 | 0.72069 |
| 127 | SAA2-SAA4 readthrough OS=Homo sapiens OX=9606 GN=SAA2-SAA4 PE=4 SV=1 | 0.47112 |
| 128 | Keratin, type II cytoskeletal 1 OS=Homo sapiens OX=9606 GN=KRT1 PE=1 SV=6 | 0.47816 |
| 129 | APOC4-APOC2 readthrough (NMD candidate) OS=Homo sapiens OX=9606 GN=APOC4-APOC2 PE=1 SV=1 | 3.36163 |
| 130 | Immunoglobulin heavy variable 3-72 OS=Homo sapiens OX=9606 GN=IGHV3-72 PE=3 SV=1 | 0.34032 |
| 131 | Biotinidase OS=Homo sapiens OX=9606 GN=BTD PE=1 SV=2 | 0.11461 |
| 132 | Uncharacterized protein (Fragment) OS=Homo sapiens OX=9606 PE=1 SV=1 | 0.63681 |
| 133 | Kallistatin OS=Homo sapiens OX=9606 GN=SERPINA4 PE=1 SV=3 | 0.23049 |
| 134 | Ficolin-3 OS=Homo sapiens OX=9606 GN=FCN3 PE=1 SV=2 | 0.41761 |
| 135 | Apolipoprotein L1 OS=Homo sapiens OX=9606 GN=APOL1 PE=1 SV=5 | 0.45364 |
| 136 | Immunoglobulin kappa variable 3-20 OS=Homo sapiens OX=9606 GN=IGKV3-20 PE=1 SV=2 | 0.02543 |
| 137 | Immunoglobulin kappa variable 1-17 OS=Homo sapiens OX=9606 GN=IGKV1-17 PE=1 SV=2 | 0.55879 |
| 138 | Immunoglobulin heavy variable 1-2 OS=Homo sapiens OX=9606 GN=IGHV1-2 PE=1 SV=2 | 0.03146 |
| 139 | Complement C1q subcomponent subunit C OS=Homo sapiens OX=9606 GN=C1QC PE=1 SV=3 | 0.16503 |
| 140 | Coagulation factor XII OS=Homo sapiens OX=9606 GN=F12 PE=1 SV=3 | 0.26835 |
| 141 | Sex hormone-binding globulin OS=Homo sapiens OX=9606 GN=SHBG PE=1 SV=1 | 0.34239 |
| 142 | Immunoglobulin heavy variable 3-9 OS=Homo sapiens OX=9606 GN=IGHV3-9 PE=1 SV=2 | 0.85246 |
| 143 | Immunoglobulin lambda constant 2 OS=Homo sapiens OX=9606 GN=IGLC2 PE=1 SV=1 | 0.63898 |
| 144 | Alpha-actinin-1 OS=Homo sapiens OX=9606 GN=ACTN1 PE=1 SV=1 | 0.43165 |
| 145 | Apolipoprotein C-I (Fragment) OS=Homo sapiens OX=9606 GN=APOC1 PE=1 SV=1 | 0.18232 |
| 146 | Apolipoprotein M OS=Homo sapiens OX=9606 GN=APOM PE=1 SV=2 | 0.51626 |
| 147 | Immunoglobulin kappa variable 3-15 OS=Homo sapiens OX=9606 GN=IGKV3-15 PE=1 SV=2 | 0.88233 |
| 148 | Phosphatidylcholine-sterol acyltransferase OS=Homo sapiens OX=9606 GN=LCAT PE=1 SV=1 | 0.19752 |
| 149 | Immunoglobulin heavy variable 3-49 OS=Homo sapiens OX=9606 GN=IGHV3-49 PE=3 SV=1 | 1.0861 |
| 150 | Coagulation factor X OS=Homo sapiens OX=9606 GN=F10 PE=1 SV=2 | 0.2931 |
| 151 | Complement C1q subcomponent subunit B OS=Homo sapiens OX=9606 GN=C1QB PE=1 SV=1 | 0.41123 |
| 152 | Complement C1r subcomponent-like protein OS=Homo sapiens OX=9606 GN=C1RL PE=1 SV=2 | 0.29641 |
| 153 | Immunoglobulin kappa variable 2-40 OS=Homo sapiens OX=9606 GN=IGKV2-40 PE=1 SV=1 | 0.58103 |
| 154 | Complement C4-B OS=Homo sapiens OX=9606 GN=C4B PE=1 SV=2 | 1.2738 |
| 155 | Fibulin-1 OS=Homo sapiens OX=9606 GN=FBLN1 PE=1 SV=1 | 0.52508 |
| 156 | Immunoglobulin lambda variable 1-47 OS=Homo sapiens OX=9606 GN=IGLV1-47 PE=1 SV=2 | 0.16442 |
| 157 | Immunoglobulin lambda variable 3-25 OS=Homo sapiens OX=9606 GN=IGLV3-25 PE=1 SV=2 | 0.34948 |
| 158 | Immunoglobulin kappa variable 4-1 OS=Homo sapiens OX=9606 GN=IGKV4-1 PE=1 SV=1 | 0.92871 |
| 159 | Complement factor H-related protein 1 OS=Homo sapiens OX=9606 GN=CFHR1 PE=1 SV=1 | 7.65486 |
| 160 | Immunoglobulin kappa variable 3-11 OS=Homo sapiens OX=9606 GN=IGKV3-11 PE=1 SV=1 | 0.70713 |
| 161 | Ceruloplasmin OS=Homo sapiens OX=9606 GN=CP PE=1 SV=1 | 2.00731 |
| 162 | Properdin OS=Homo sapiens OX=9606 GN=CFP PE=1 SV=1 | 0.24251 |
| 163 | Immunoglobulin heavy variable 4-31 OS=Homo sapiens OX=9606 GN=IGHV4-31 PE=3 SV=1 | 0.58465 |
| 164 | Keratin, type II cytoskeletal 2 epidermal OS=Homo sapiens OX=9606 GN=KRT2 PE=1 SV=2 | 0.12159 |
| 165 | Apolipoprotein(a) OS=Homo sapiens OX=9606 GN=LPA PE=1 SV=1 | 0.44232 |
| 166 | Trypsin-1 (Fragment) OS=Homo sapiens OX=9606 GN=PRSS1 PE=1 SV=1 | 0.67322 |
| 167 | Immunoglobulin lambda variable 6-57 OS=Homo sapiens OX=9606 GN=IGLV6-57 PE=1 SV=2 | 0.40109 |
| 168 | Complement C2 OS=Homo sapiens OX=9606 GN=C2 PE=1 SV=2 | 0.22813 |
| 169 | C-reactive protein OS=Homo sapiens OX=9606 GN=CRP PE=1 SV=1 | 0.2505 |
| 170 | Immunoglobulin kappa variable 1-9 OS=Homo sapiens OX=9606 GN=IGKV1-9 PE=3 SV=1 | 1.13737 |
| 171 | IgGFc-binding protein (Fragment) OS=Homo sapiens OX=9606 GN=FCGBP PE=1 SV=2 | 0.59984 |
| 172 | Immunoglobulin lambda constant 7 OS=Homo sapiens OX=9606 GN=IGLC7 PE=1 SV=3 | 0.21862 |
| 173 | Immunoglobulin heavy variable 3-74 OS=Homo sapiens OX=9606 GN=IGHV3-74 PE=3 SV=1 | 1.12607 |
| 174 | Immunoglobulin lambda variable 5-45 (Fragment) OS=Homo sapiens OX=9606 GN=IGLV5-45 PE=1 SV=1 | 1.48674 |
| 175 | Immunoglobulin heavy variable 3-13 OS=Homo sapiens OX=9606 GN=IGHV3-13 PE=1 SV=2 | 1.19729 |
| 176 | Serum amyloid A protein OS=Homo sapiens OX=9606 GN=SAA1 PE=1 SV=1 | 0.17709 |
| 177 | Immunoglobulin heavy variable 3/OR16-12 (non-functional) (Fragment) OS=Homo sapiens OX=9606 GN=IGHV3OR16-12 PE=1 SV=1 | 0.21873 |
| 178 | Immunoglobulin heavy variable 4-28 OS=Homo sapiens OX=9606 GN=IGHV4-28 PE=3 SV=1 | 0.49811 |
| 179 | Bridging integrator 3 OS=Homo sapiens OX=9606 GN=BIN3 PE=4 SV=1 | 0.60121 |
| 180 | Rho-associated protein kinase 1 OS=Homo sapiens OX=9606 GN=ROCK1 PE=1 SV=1 | 0.74277 |
| 181 | Apolipoprotein C-IV OS=Homo sapiens OX=9606 GN=APOC4 PE=1 SV=1 | 0.43824 |
| 182 | Protein Smaug homolog 2 OS=Homo sapiens OX=9606 GN=SAMD4B PE=1 SV=1 | 0.42571 |
| 183 | Probable helicase with zinc finger domain OS=Homo sapiens OX=9606 GN=HELZ PE=1 SV=1 | 1.17979 |
| 184 | Integrin beta OS=Homo sapiens OX=9606 GN=ITGB3 PE=3 SV=1 | 0.26295 |
| 185 | Beta-2-microglobulin (Fragment) OS=Homo sapiens OX=9606 GN=B2M PE=1 SV=1 | 2.58333 |
| 186 | Immunoglobulin lambda variable 10-54 OS=Homo sapiens OX=9606 GN=IGLV10-54 PE=1 SV=1 | 0.27839 |
| 187 | Transcription factor Sp5 OS=Homo sapiens OX=9606 GN=SP5 PE=2 SV=1 | 1.1819 |
| 188 | Protein BEX5 OS=Homo sapiens OX=9606 GN=BEX5 PE=1 SV=1 | 0.34112 |
| 189 | Immunoglobulin heavy variable 6-1 OS=Homo sapiens OX=9606 GN=IGHV6-1 PE=3 SV=1 | 0.49616 |
| 190 | Cadherin-5 OS=Homo sapiens OX=9606 GN=CDH5 PE=1 SV=5 | 0.13965 |
| 191 | Immunoglobulin heavy variable 3-43 OS=Homo sapiens OX=9606 GN=IGHV3-43 PE=3 SV=1 | 0.25822 |
| 192 | Immunoglobulin kappa variable 3D-20 OS=Homo sapiens OX=9606 GN=IGKV3D-20 PE=3 SV=1 | 0.57388 |
